# Preventing adolescent sexual harassment: evaluating the planning process in two school-based interventions using the Intervention Mapping framework

**DOI:** 10.1186/s12889-019-7808-8

**Published:** 2019-11-06

**Authors:** Gaby P. A. de Lijster, Gerjo Kok, Paul L. Kocken

**Affiliations:** 1 TNO, PO Box 3005, Leiden, 2301 DA the Netherlands; 2 Maastricht University, PO Box 616, Maastricht, 6200 MD the Netherlands; 3 Department of Public Health and Primary Care, Leiden University Medical Centre (LUMC), PO Box 9600, Leiden, 2300 RC the Netherlands

**Keywords:** Prevention, Adolescent sexual harassment, Practice-based interventions, Intervention mapping Planning process

## Abstract

**Background:**

The development of school-based programs for preventing adolescent sexual harassment often lacks an evidence-based approach and use of proper theories. Appropriate stakeholders are often not involved in the development process. To help improve this process, we used the Intervention Mapping framework to retrospectively evaluate the development of two school-based programs, *Benzies & Batchies* and *Boys*, each of which was intended to prevent sexual harassment among adolescent students of a lower educational level in the Netherlands. The two interventions were among the first school-based programs targeting sexual harassment, and were implemented in Dutch secondary schools.

**Methods:**

As well as doing desk research into the context and content of the interventions, we used semi-structured focused interviews with the initial developers to gather their opinions on and experiences with the development process, whereby the topics were based on the six steps of the IM framework. To better suit the needs of the respondents, we had adapted the language of our topics and had used open-ended questions The data we had gathered from the desk research and face-to-face consultations were checked against a planning tool that was based on 19 tasks within the six steps of IM.

**Results:**

Although both programs had been developed in practice and lacked a thorough theoretical foundation, the methods and materials used represented aspects of behavior-change theories. The developers of *Benzies & Batchies* completed slightly more planning criteria within the six steps of the planning process, and used more change methods than the developers of *Boys* did.

**Conclusions:**

We recommend that parents should also be involved in the development of sex and relationship education programs, and should be allowed to participate in the program itself. To meet the needs of intervention developers, greater insight is needed into the importance of the individual steps in the Intervention Mapping framework. In our view, the development of practice-based interventions will improve if future intervention developers combine evidence-based theories with their practice-based experience. This will increase the success and effectiveness of their interventions.

## Background

When it comes to their sexual health, adolescents around the world are faced with various challenges, such as teenage pregnancy and sexually transmitted infections [1]. There has also been an increase in adolescent sexual harassment behaviors, such as sending and receiving nonconsensual sexually tinted messages (so-called ‘sexting’) [2]. Recent research shows a higher mean prevalence rate of 27% for receiving nonconsensual sexual messages compared to 15% of sending such messages [2]. Besides, Wincentak and colleagues recently reported an overall prevalence of 9% when it comes to sexual teen dating violence [3].

After undergoing sexual harassment, young people in general, and vulnerable girls and boys in particular, are at risk for short and long term health problems [4]. Various school-based programs therefore attempt to prevent sexual harassment. These programs differ in various ways, especially with regard to their methods and targeted outcomes, and also to the age and gender of their target populations [5].

In their analysis of 55 qualitative studies among young people aged between 12 and 18 years in 10 different countries, Pound and colleagues [6] concluded that school-based sex and relationship education did not meet the needs of the students receiving it. A second problem is that preventive programs paid little attention to sexual harassment [7]; a third is that there are few effective school-based programs on this topic [8].

**Fig. 1:**
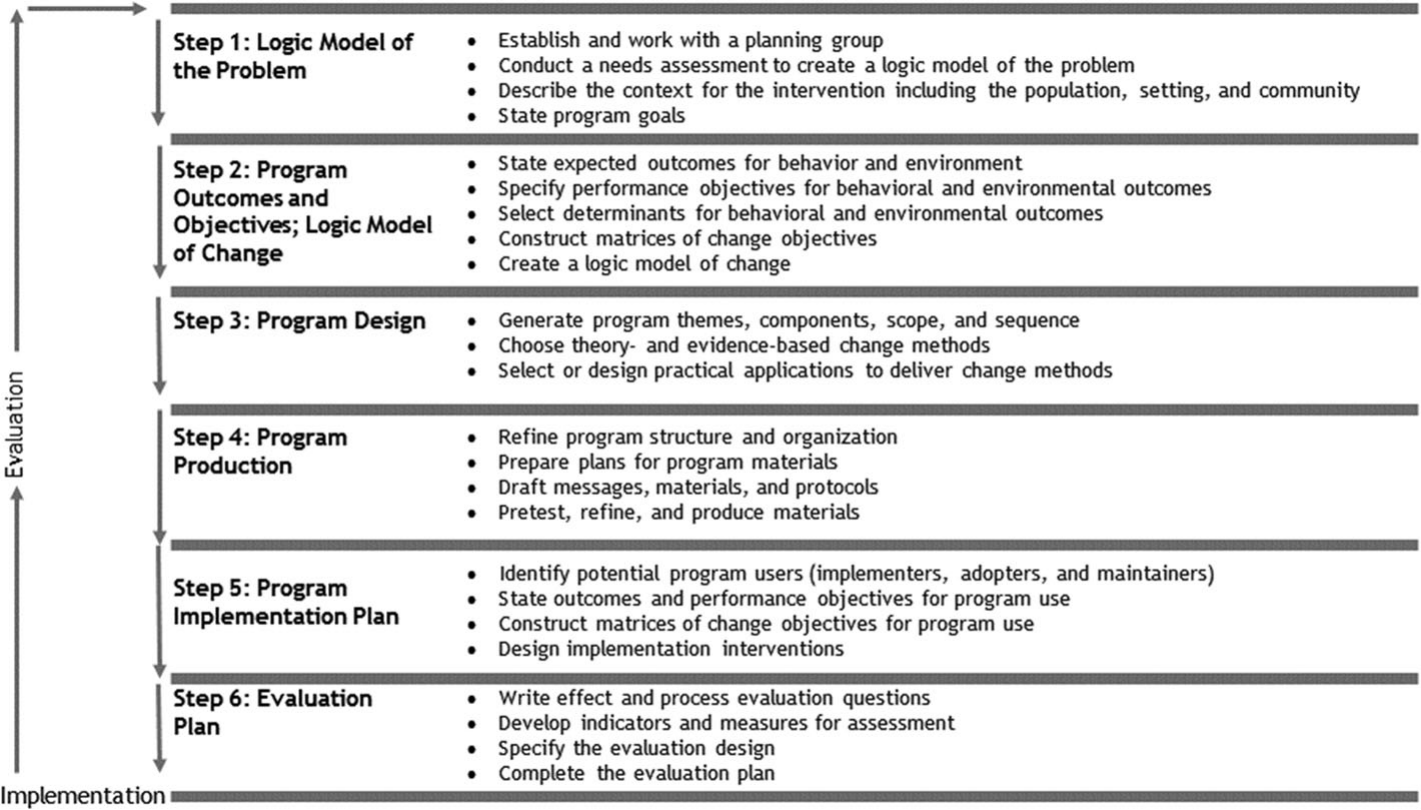
The six steps of Intervention Mapping [10]

School-based programs are often developed by professionals in practice, and often lack the systematic and planned development that is a precondition for success [9]. And if one wishes to review the theoretical basis of a sexual health program, one needs a systematic description not only of the context and content of the intervention, but also of its objectives, its performance and change objectives, and its change methods [10]. Such a description can be provided by Intervention Mapping (IM), a six-step framework that enables those planning, developing, implementing and evaluating health-promotion programs to make effective decisions at each step [10] (see Fig. 1).

The objective of IM step 1 is to conduct a needs assessment. This will make it possible to develop a logic model of the problem, i.e., of the factors that cause or influence the health problem on which the intervention will focus. The goal of step 2 is to develop a logic model of change, in which program planners specify what needs to change in behavior and the environment in order to improve health and quality of life. The goal of step 3 is to design the program on the basis of theory and evidence. The goal of step 4 is to produce the health-promotion program by building on the input gathered in the first three steps. The goal of step 5 is to develop an implementation plan for its adoption, implementation and maintenance. In the sixth and final step of the IM process, program planners develop and complete an evaluation plan. Within each of the six steps, several tasks must be completed in order to move on to the next step in the process. At the same time, this creates feedback loops. If the program planner takes proper account of these feedback loops, the intervention is more likely to be matched to the needs of the target group [11].

The IM framework enables program planners to make effective decisions during the planning process. Using its six steps, Godin and colleagues [12] developed a planning tool that enables professionals to evaluate the potential success of an intervention by analyzing the rigor with which it was developed. The tool thus helps to the identify strengths and weaknesses of its planning.

The objective of this study was to use the Intervention Mapping framework [10, 12] to retrospectively evaluate the strengths and weaknesses of the planning process of two interventions *Benzies & Batchies* and *Boys*. These two existing school-based programs were aimed at the prevention of sexual harassment among adolescents. The target groups for these programs were adolescents attending schools for prevocational secondary education. The first program, *Benzies & Batchies*, which was first given in 2011, targeted both boys and girls, and addressed the harassment behaviors from both the victim’s and the perpetrator’s perspectives [13]. The second, *Boys*, which was first given in 2008, targeted adolescent males only, and stressed the perspective of the male perpetrator [14]. The two interventions were among the first school-based programs targeting sexual harassment, and were implemented in Dutch secondary schools. They were selected for this paper due to their innovative approach to educating adolescents at a lower educational level on sex and relationship skills. Each was an example of good practice and had been included in a national program of promising interventions for developing and evaluating sexual health programs for adolescents.

While each intervention had been developed by professionals in practice, little was known about the development process or the theoretical rationale underlying it. We therefore wished to evaluate the strengths and weaknesses of *Benzies & Batchies* and *Boys* and thereby to contribute to the development of practice-based interventions. Specifically, we wished to establish the extent to which the development, including implementation plan and evaluation plan, had followed a planned process. Taking the six steps of the Intervention Mapping protocol as our developmental criterion, we also wished to establish which steps had been carried out in the process of developing these interventions [12, 15].

## Methods

We combined various research methods for the retrospective study of the process whereby *Benzies & Batchies* and *Boys*, two school-based interventions for preventing adolescent sexual harassment, had been developed.

### Desk research

We first conducted desk research into the context and content of the interventions. To this end, we consulted the interventions’ websites and studied the intervention materials available. The theatre play that was part of *Benzies & Batchies* comprised short scenes in which male and female peer-educators performed examples of sexual harassment (both victimization and perpetration) and of reactions to them. The play was followed by a group discussion led by the peer-educators. The students were then given a skills and resilience training. *Boys* consisted of five consecutive lessons intended to teach skills with regard to social and sexual behavior and to set standards for appropriate sexual behavior by boys. For an overview of the context and content of *Benzies & Batchies* and *Boys*, see Table 1.

**Table 1:** Overview of the context and content of *Benzies & Batchies* and *Boys*

Intervention	Component	Duration	Content	Giver	Receiver	Setting
*Benzies & Batchies*	Introductory lesson	50 min	Letter for teacher	Teacher	Boys and girls, 12–14 years	Classroom
	Peer-performed theatre play, followed by group discussion	30 + 60 min	Script	Peer-educators		Auditorium
	Skills and resilience training	Three 100–150-min lessons	Student workbook	Trained social skills instructor		Classroom
	Closing lesson	50 min	Letter for teacher	Teacher		Classroom
*Boys*	Classroom lesson	Five 50-min lessons (basic lesson)	Box containing:	Trained male instructor	Boys, 12–14 years	Classroom
			- teacher’s manual			
			- DVD			
			- worksheets			

### Semi-structured focused interviews

Next, we carried out semi-structured focused interviews with the developers of the two interventions to collect their opinions on the initial development process [15]. These focused interviews took place at the offices of the intervention owners. For the two interventions, we carried out three interviews with a total of four participants. Two researchers (GL and PK) carried out one interviews with two participants, i.e., the initial developer of *Benzies & Batchies*, and a program trainer; and one researcher (GL) carried out two interviews, each with one of the initial developers of *Boys.* We based our topics for the interviews on the six steps of the IM framework, adapting the language of our topics to better suit the needs of the participants, and using open-ended questions [10]. Each focused interview lasted about one hour. See Table 2 for an overview of the topics.

**Table 2:** Overview of topics

IM Step 1: logic model of the problem	
1	What was the reason for developing the program?
2	What was the base for the development of the program?
2a	Who was involved in the early development of the program?
3	On which health problem/health behavior does the program focus?
3a	Which factors influence the health problem/health behavior?
3b	How was the need for the program determined?
4	For whom is the program intended?
IM Step 2: logic model of change	
1	What is the main objective of the program?
1a	What are the objectives of the different lessons/parts of the lessons?
2	What change is expected?
2a	To what, or to whom?
2b	Are the expected changes formulated in terms of outcomes?
IM Step 3: program design	
1	Was the target group involved in the compilation of the program or parts of it?
2	On which theory is the intervention, or parts of it, based?
3	Which methods are used in the program and why?
4	Which strategies are used in the program and why?
5	Which materials are used in the program?
IM Step 4: program production	
1	Was the target group involved in the design of the program?
2	Were all the objectives that were chosen beforehand included in the program?
3	Were the methods, strategies and materials chosen tested beforehand?
4	To what extent does the program fit specific needs encountered in practice (i.e., at school)?
5	Was the program, or were parts of it, adapted during the program?
IM Step 5: adoption and implementation	
1	Were barriers to the implementation of the program envisaged?
2	Was the program implemented in its entirety?
3	To have the desired effect on the students, what is the minimum number of lessons or parts of lessons that need to be performed?
4	Are schools supported in carrying out the program? How?
5	Can the quality of any of the program be guaranteed?
IM Step 6: evaluation	
1	Is the program evaluated during and/or at the end of the program?
2	Who is involved in the evaluation?

### Planning tool

Finally, we checked the data we had gathered from the desk research and focused interviews against a planning tool that was based on the 19 tasks within the six steps of IM (see Table 3). This planning tool consisted of 40 planning criteria that had been identified in an earlier study by experts from community, public health, and university environments [12]. By completing this tool we evaluated the extent to which the program planners had done the following: created a logic model of the problem that included a clear statement of the problem and problem owner (IM Step 1; 4 tasks; 9 criteria); created a logic model of change that included a clear statement of the changes in behavior and environment that were needed to accomplish improvements in health outcomes (IM Step 2; 5 tasks; 11 criteria); designed the program with practical applications for delivering the theory-based and evidence-based change methods of their choice (IM Step 3; 2 tasks; 4 criteria); structured the program, and produced the materials; and used the information gathered in steps 1 to 3 as input (IM Step 4; 3 tasks; 10 criteria); developed an implementation plan (IM Step 5; 1 task; 2 criteria); and finally, had developed an evaluation plan (IM Step 6; 4 tasks; 4 criteria).

**Table 3:** Planning criteria – overview of tasks, criteria and results per intervention

Task	Criterion	Benzies & Batchies	Boys
		accomplished	accomplished
	IM Step 1: logic model of the problem		
	*Identify the problem*		
1	Consult literature	–	–
2	Validate with local supporters	+	+
	*Identify the target population*		
3	Socio-demographic profile	+/−	+/−
4	Socio-cultural context	+/−	+/−
	*Identify determinants*		
5	Consult literature	–	–
6	Gather information on the population	+/−	+/−
	*Analyze the environment*		
7	Identify places, methods and times to contact the participants	+	+
8	Identify hindering and facilitating factors	+	+
9	Identify partners and their respective roles	+	+
	IM Step 2: logic model of change		
	*Specify the population*		
10	Consider particularities	+	+
	*Overall objective*		
11	Word precisely (targeted change)	+	+
	*Performance objectives*		
12	Specify what should be obtained	+	+
13	Develop objectives based on theory, empirical data or deep understanding	–	–
14	Validate with partners	–	–
	*Choice of determinants*		
15	Choose with respect to their connection with the targeted behavior	+/−	–
16	Choose with respect to their potential success	–	–
17	Validate with partners	–	–
	*Learning objectives*		
18	Related to performance objectives and determinants	–	–
19	Based on theoretical notions	–	–
20	Validate with partners	–	–
	IM Step 3: program design		
	*Choose the models*		
21	Support with tested theoretical methods	–	–
22	Consider population characteristics	+	+/−
	*Translate into strategies*		
23	Support with theory	–	–
24	Validate with partners	+/−	–
	IM Step 4: program production		
	*Organizational structure*		
25	Consider limitations of the milieu	+	+
26	Carry out with partners	+	+
27	Train and support workers	+	+
	*Sequence and content of activities*		
28	Activities related to objectives	+	+
29	Realistic calendar	+	–
30	Validate with partners	+	–
	*Production of material*		
31	Involvement of partners	+	+
32	Begin scheduled activities	+	+
33	Accessible and properly communicated	+	+
34	Adapt the material	+	+
	IM Step 5: adoption and implementation		
	*Support of decision-makers and community*		
35	Active partners	+	+/−
36	Identify the person in charge	+	+
	IM Step 6: evaluation		
	*Evaluation plan*		
37	Plan before implementation	+/−	–
	*Process*		
38	Document information about the population and the intervention	+	+
	*Impact*		
39	Measure the degree to which objectives are achieved	+	+
	*Communication*		
40	Discuss findings with partners	+	+/−

+ accomplished

+/− partially accomplished

– not accomplished

### Data analysis

First, working independently of each other, two researchers (GL and PK) rated the degree of project planning of each intervention by coding each of the 40 criteria within the 19 tasks of the six steps of IM: “+” fully accomplished; “+/-“ partially accomplished; “-“ not accomplished. In case of differences between the findings of the two researchers, the results of the coding were discussed until both researchers agreed on the appropriate coding. Next, for each intervention, the degree of project planning within each task was analyzed by calculating the overall score per task, whereby the total number of observable criteria per task was summed. Tasks were considered to have been accomplished if at least one planning criterion for each task was found to have been “fully accomplished”. If half of the tasks within the step had been completed properly, we considered the planning of each of the six IM steps to have been carried out [12].

## Results

### Development and planning process

(Also see also Table 3).

#### IM step 1: logic model of the problem

Within the first step of IM, the initial intervention developers needed to have established and worked with a planning group that included various stakeholders, such as community members, potential program implementers and program beneficiaries. The context of the intervention, including its population, setting and community, also needed to have been described.

During the focused interviews, the developers of both programs had stated clear reasons for developing their program and had clearly identified their target groups. The initial developers of *Benzies & Batchies* had noticed the influence of R&B video clips on the contact between young girls and older boys, for example at parties. *Benzies & Batchies* was developed out of a need to develop a program that focused on the relational side of sex rather than on the factual and technical side, such as on sexually transmitted infections. The initial developers of *Boys* had realized that joint male and female education on sex and relationships did not always meet students’ and schools’ wishes, and preventive school-based programs for perpetrators of sexual harassment – most of whom were boys – were missing. The *Boys* program was developed out of a need to address not only the victims of sexual harassment but also the perpetrators. Both interventions had been developed from the developer’s point of view and pilot-tested for their target group. Subsequently, program beneficiaries including adolescents, peer educators and teachers had been involved in the further development of the programs, and also professional skills trainers with regard to the development of the skills training. Other stakeholders - such as community members (e.g., parents), potential program implementers (e.g., school board members) or behavioral scientists - had not been included in the planning groups.

Two of four tasks pertaining to step 1 had been fully accomplished for both programs (see Table 3). As a consequence, the planning process of IM Step 1 had been carried out.

#### IM step 2:logic model of change

Within the second step of IM, the initial intervention developers needed to have developed a logic model of change, and to have specified what needed to change with regard to behavior and the environment for improvements in health and quality of life to take place.

According to their developers, the main objective of their programs had been to prevent adolescent sexual harassment behavior (or, in other words to reduce risk behavior); in the *Boys* program, the particular target had been sexual harassment behavior in male students. During the focused interview the developer of *Benzies & Batchies* stated that creating awareness, being able to state one’s personal boundaries and dealing with peer pressure had been program outcomes of the theatre play. Working on social skills, sexual self-esteem and self-efficacy were the stated outcomes of the social skills training. The developers of the *Boys* program also mentioned creating awareness, being able to state one’s personal boundaries, and dealing with peer pressure as outcomes of their lesson series.

From the desk research and the focused interviews it became apparent that although the developers of the two programs had mainly used practice-based evidence to develop their logic model of change, they had not consulted the scientific literature. Consequently, they were unable to specify the theories underlying their programs. By reconstructing the answers given during the interviews, we were able to trace theories at the individual level. These included the theories of reasoned action (i.e., Reasoned Action Approach [16]) and persuasive communication (i.e., Communication-Persuasion Matrix [17]; Elaboration Likelihood Model [18]). At an interpersonal level, they included Social Cognitive Theory [19]. While both developers mentioned outcomes that focused on the individual and interpersonal level, no outcomes had been formulated at organizational, community or societal levels.

Of the five tasks pertaining to step 2, three had been fully accomplished for each intervention (see Table 3). Consequently, the planning process of IM Step 2 had been carried out.

#### IM step 3: program design

Within the third step of IM, the initial intervention developers needed to have designed their program by generating program themes, program components and program scope and sequence by choosing theory- and evidence-based change methods and by selecting or designing practical applications for delivering change methods (see Tables 1 and 4).

**Table 4:** Overview of change methods

		Benzies & Batchies	Boys
		used in the program	
Basic methods at the individual level			
• Persuasive communication	Use arguments to guide students toward an attitude or action	√	√
• Active learning	Encourage students to learn from activity-based experience	√	√
• Modeling	Provide an appropriate model	√	√
• Feedback	Give the students information on the extent to which they are accomplishing learning or performance	√	√
Methods to change attitudes, beliefs			
• Self-reevaluation	Encourage students to combine cognitive and affective assessments of their self-image with and without the desired behavior	√	–
• Arguments	Use a set of one or more meaningful premises and a conclusion	√	–
Methods to change social influence			
• Resistance to social pressure	Stimulate students to build skills for resisting social pressure	√	–
Methods to change skills, capability and self-efficacy			
• Guided practice	Prompt students to rehearse and repeat the behavior various times, discuss their experiences, and provide feedback	√	–
• Verbal persuasion	Use messages that suggest that the student possesses certain capabilities	√	–
• Planning coping responses	Prompt students to list potential barriers and ways to overcome them	√	√
Methods to increase knowledge			
• Discussion	Encourage consideration of a topic by the students in an open informal debate	√	√
• Elaboration	Stimulate the student to add meaning to the information that is processed	√	√
Methods to change social norms			
• Entertainment education	Provide a form of entertainment designed both to educate on sexual behavior and to entertain	√	–
Methods to change social support and social networks			
• Peer-education	Mobilize members of the target population to serve as credible sources of information and role models	√	–

As with the construction of the logic model of change (see IM Step 2) it was obvious from the desk research and the focused interviews that the developers of both programs had mainly used practice-based evidence to design their programs. However, by using peer-educators who acted as role models in the theatre play for the adolescent target group, the developer of *Benzies & Batchies* had incorporated a theory- and evidence-based change method. On the basis of their experience, the developers of the *Boys* program had chosen to use various applications that fitted the adolescent male target group (see Table 4).

Of the two tasks pertaining to step 3, one task had been fully accomplished for *Benzies & Batchies*. The planning process of IM Step 3 had thus been carried out. In the *Boys* program, the planning process of IM Step 3 had not been carried out, as no tasks pertaining to this step had been fully accomplished (see Table 3).

#### IM step 4: program production

Building on the input gathered in steps 1–3, the initial intervention developers needed to have produced the programs within the fourth step of IM. They thus needed to have refined the program structure and organization, to have prepared plans for program materials, to have drafted messages, materials and protocols, and to have pretested, refined and produced materials.

For *Benzies & Batchies* and for *Boys*, all three tasks pertaining to step 4 had been fully accomplished. The planning of this step in the Intervention Mapping process had thus been carried out (see Table 3). Both developers stated that they had used individuals from their target group to pretest the materials and had then adapted these materials on the basis of these individuals’ experiences. On the basis of the pretest, future users of *Benzies & Batchies* had been recommended to carry out the complete program in order to accomplish the specified goals, and to achieve lasting effects on behavior. In addition, based on the peer-educators’ personal experiences with sexual (harassment) behavior, (media) trends and results from national research on the topic an update for the basic script of the theatre play was provided every year.

It was clear from the interviews with the developers of *Boys* that the structure, organization, messages and materials of the program had been based only on practice and experience. The developer clarified that they had added an optional five extra lessons to the basic lessons from the beginning because there were too many topics and methods to accommodate within the basic lessons. They had made a critical selection of the topics and methods based on the goals of each lesson, and had distributed the topics and methods over the basic and extra lessons whereby goals should be achieved by carrying out the five basic lessons.

#### IM step 5: adoption and implementation

Throughout the development process, but specifically in step 5 of IM, the initial intervention developers needed to have developed an implementation plan for adopting, implementing and maintaining the program.

The developers of the program *Benzies & Batchies* had developed an implementation manual that contained detailed information on the proper performance and maintenance of their program. Several times a year, newsletters and promotion folders were sent to schools. One person was responsible for press policy. The developers stated that, to provide continuity in guidance on the theme, they had established collaborative relationships with regional specialist organizations such as the addiction and municipal welfare services. Consultation took place with municipalities about embedding *Benzies & Batchies* in their policies and implementation plans.

Although no implementation plan was available for the program of *Boys* the developers stated they had recruited schools for participation in the program mainly in a large city in the Netherlands whose city council had also provided structural funding. Funding for the program was sought together with schools in other areas.

IM Step 5 consisted of one task: ‘Support of decision-makers and community’, which had been fully accomplished for both interventions. This step had thus been carried out (see Table 3).

#### IM step 6: evaluation

Throughout the development process, but specifically in the sixth step of IM, the initial intervention developers needed to have developed and completed an evaluation plan by doing the following: writing questions for effect and process evaluation, developing indicators and measures for assessment, specifying the evaluation design, and completing the evaluation plan.

In the focused interviews, the developers of the two interventions stated that they had not themselves developed and completed an evaluation plan. However, they had constructed a limited evaluation procedure and an independent research institute had conducted effect evaluations of the programs [20, 21]. The developers of *Benzies & Batchies* had arranged for annual internal evaluation with the peer-educators who had performed the theatre play and had led the group discussion afterwards. In addition, where relevant, the students’ comments and experiences were discussed with the students’ teachers or with members of the school board. The developers of *Boys* had compiled a short eight-item questionnaire on their lessons, which had been completed by the male students at the end of the lesson series. The developer used the results of these questionnaires as an internal review procedure for the trainers. Afterwards, the intervention developers of both programs adapted their programs on the basis of the effect evaluations and of the recommendations that followed from them.

Of the four tasks pertaining to step 6, three tasks for *Benzies & Batchies*, and two tasks for *Boys* had been fully accomplished (see Table 3). The planning process of IM Step 6 had thus been carried out.

## Discussion

To improve the development of such practice-based interventions, we evaluated the strengths and weaknesses of the planning process of two school-based preventive sexual-harassment interventions, *Benzies & Batchies* and *Boys*.

After using a planning tool to retrospectively assess the accomplishment of the Intervention Mapping process we concluded that all six IM steps had been carried out for *Benzies & Batchies* and that all but one step had been carried out for *Boys*. However, for both interventions, we found that the intervention developers had carried out tasks within the steps concerning the programs’ production, adoption, implementation and evaluation but less within the steps regarding the needs assessment and theoretical underpinning of the programs. Our findings show that although both programs lacked a thorough theoretical foundation (as suggested within IM Steps 1, 2, and 3), the methods and materials that were used represented aspects of evidence-based behavior change theories. The intervention developers made limited descriptions of the problem and their target groups, they did not consult the literature on the prevalence of the problem or on the disease burden, and neglected to examine whether the objectives they specified were feasible.

When developing a program, a range of benefits follow from including all IM steps. If each task is performed per step, and each step is thus completed, the intervention will have a proper theoretical underpinning. This will lead to effective decisions on its development, implementation and evaluation. The Intervention Mapping framework may be considered to be a rigid approach to developing an intervention. As the first two first steps of the IM protocol are complex, time-consuming and costly, intervention developers sometimes choose to modify the original IM protocol or not to perform all tasks per step [11, 22]. In this respect the results of the present study are consistent with those found in a study that explored the development of existing sex-education programs for people with intellectual disabilities [15].

The initial developers of the two interventions had used a range of change methods that focused on determinants of behavior, with the developers of *Benzies & Batchies* using twice as many methods as the developers of *Boys*. More practical applications had also been used in the *Benzies & Batchies* program, including a peer-performed theatre play, group discussions and skills training, than in the *Boys* program which focused mainly on group discussions and on assignments using worksheets. Using a range of change methods and practical applications may lead an intervention to have more positive outcomes. In earlier interventions, for example, the combination of a classroom intervention with a broader environmental intervention proved to be more effective in reducing adolescent dating violence and sexual harassment than the use of the single classroom intervention [23]. Similarly, multicomponent school interventions were effective for promoting adolescent sexual health [24]. Effective school-based interventions targeting physical activity among older adolescents were also found to have used more behavior-change techniques than non-effective interventions [25].

The systematic and planned development of an intervention may contribute to its success and its effects on health outcomes [26]. Effect evaluations of *Benzies & Batchies* and *Boys* [20, 21] showed mixed results. Whereas significant short and long term effects on the determinants of adolescent sexual harassment behavior were found for *Benzies & Batchies*, no significant effects were found for the *Boys* program. As the results of the present study show that the developers of *Benzies & Batchies* had met slightly more planning criteria within the six steps of the planning process and had used more change methods than the developers of *Boys*, we suggest that an intervention produces better results if a planned approach has been taken to its development.

### Strengths and limitations

Our use of various research methods enabled us to consult different sources with regard to the process whereby the interventions were planned. However, the focused interviews with the initial intervention developers took place several years after the initial development. As a result, the respondents may have had difficulty in thinking back to the period in question. In addition, both interventions had gradually been adjusted in the period between initial start of the intervention and time of the interview which means that our research was a reflection of the moment. As a development process is not static, this may be seen as a limitation. For the same reason, it may also be seen as a strength, as developers can move back and forth between the tasks within the IM steps, using the information they gather to gradually adapt their intervention [10]. As insight increases, an intervention can develop.

We used the Intervention Mapping framework to establish the extent to which the two interventions had been planned. This framework was developed as an approach to systematically plan the process of development of health-promotion programs. Although it has not been directly compared to other processes for developing interventions, it is widely used today [10]. Lastly, when completing the planning tool to evaluate the interventions, we found it difficult to complete all the entries, as the tool was not provided with instructions or further explanation of the planning criteria. This might have led to overestimation or underestimation in the subjective scoring of the planning criteria by the researchers.

### Implications for practice

First of all, the intervention developers had involved teachers and students in the development of their programs. Previous research showed that involving them in the development and evaluation of school-based sex education programs was effective in the later adoption of the program [27, 28]. In line with earlier research results [24, 29–31], we recommend that parents should also be involved in the development of programs focusing on adolescent sexual health. And to provide a connection with the education on sex and relationships that parents give their children, we recommend that parents are also allowed to participate in the programs. Involving them in program development helps identify topics the program should include [29]. Allowing them to participate in the program through family homework assignments helps them to share their norms and values with their children [31]. Boys in particular can benefit from parental involvement, as conversations about sex and sexual behavior may start earlier and occur more frequently, which may in turn causes them to delay sexual intercourse [30].

Second, intervention developers need to combine their practice-based experience with evidence-based theories, either by means of consulting scientific literature themselves or by working together with researchers in the planning group. This synergy will lead to more successful and effective interventions.

### Recommendations for further research

To date, the Intervention Mapping framework has been used to develop many interventions focusing on various health outcomes [32]. Not all have been evaluated and results on health outcomes are mixed [28]. Many interventions have also been developed on the basis of professionals’ practical experience [15]. To meet their needs, greater insight is needed into the importance of each step in the IM framework. Is each task within an IM step as important as the others? To accomplish a planned process, is it necessary to carry out each task within each step?

The developers of the two interventions had adapted their program in response to the results of the effect evaluations and to the recommendations given by researchers, for example with regard to the theatre play of *Benzies & Batchies* and the short movies on the DVD in *Boys*. We recommend further research into the effects of these adaptations, as developing an intervention according to the IM framework is an iterative and ongoing process, and changes in culture, environment and target group can have an effect on the outcomes of the intervention [10].

Besides the usefulness of the IM framework for intervention development, it is also useful for evaluating existing interventions. In comparison to other approaches and tools that exist with regard to the design process of an intervention [33], the IM framework offers an overall approach, which includes not only steps for planning, developing, and implementing an intervention, but also steps for evaluating and adapting existing health promotion programs.

## Conclusions

The two school-based programs *Benzies & Batchies* and *Boys* were both the product of incidents involving adolescent sexual harassment. Although both programs were developed in practice and lacked a thorough theoretical foundation, their methods and practical applications represented aspects of behavior-change theories. The intervention developers had completed many parts of the planning process, emphasizing the program’s production, adoption, implementation and evaluation. If future intervention developers combine their practice-based experience with evidence-based theories, the development of practice-based interventions will improve, leading to more successful and effective interventions.

## Data Availability

The datasets used and/or analysed during the current study are available from the corresponding author on reasonable request.

## Abbreviation

IM: Intervention Mapping
